# Load-sharing characteristics of stenting and postdilation in heavily calcified coronary artery

**DOI:** 10.21203/rs.3.rs-3147116/v1

**Published:** 2023-07-17

**Authors:** Pengfei Dong, Jose Colmenarez, Juhwan Lee, Neda Shafiabadi Hassani, David L. Wilson, Hiram G. Bezerra, Linxia Gu

**Affiliations:** Florida Institute of Technology; Florida Institute of Technology; Case Western Reserve University; University Hospitals Cleveland Medical Center; Case Western Reserve University; The University of South Florida; Florida Institute of Technology

**Keywords:** Calcification, stenting, radial force, equivalent pressure, ballon oversize, load transfer, post-dilation, percutaneous coronary intervention, finite element method

## Abstract

In this work, stenting in non-calcified and heavily calcified coronary arteries was quantified in terms of diameter-pressure relationships and load transfer from the balloon to the artery. The efficacy of post-dilation in non-calcified and heavily calcified coronary arteries was also characterized in terms of load sharing and the changes in tissue mechanics. Our results have shown that stent expansion exhibits a cylindrical shape in non-calcified lesions, while it exhibits a dog bone shape in heavily calcified lesions. Load-sharing analysis has shown that only a small portion of the pressure load (1.4 N, 0.8% of total pressure load) was transferred to the non-calcified lesion, while a large amount of the pressure load (19 N, 12%) was transferred to the heavily calcified lesion. In addition, the increasing inflation pressure (from 10 to 20 atm) can effectively increase the minimal lumen diameter (from 1.48 mm to 2.82 mm) of the heavily calcified lesion, the stress (from 1.5 MPa to 8.4 MPa) the strain energy in the calcification (1.77 mJ to 26.5 mJ), which associated with the potential of calcification fracture. Results indicated that increasing inflation pressure can be an effective way to improve the stent expansion if a dog bone shape of the stenting profile is observed. Considering the risk of a balloon burst, our results support the design and application of the high-pressure balloon for post-dilation.

## Introduction

Stenting in heavily calcified coronary arteries is challenging due to the inherent residual stent underexpansion and malapposition. Post-dilation with a shorter balloon at a higher inflation pressure has been widely adopted to improve the stent expansion. However, it increases the risk of intima dissection and vessel rupture [1, 2]. A retrospective study has scored stent sizing, balloon post-dilation, and pre-dilation to predict one-year adverse cardiac events following implantations of bioresorbable stent [4]. There is also mounting evidence to query if pre-dilation is necessary [5–7]. A clinical report of eight patients has demonstrated the efficacy of a noncompliant balloon with an ultra-high inflation pressure of 40 atm [3]. However, the contribution of high inflation pressure to arterial mechanics was not clear. In addition, no existing publications quantified how the inflation pressure is transferred from the balloon to the stent-lesion system. The physical characterization of stenting and post-dilation could help to address the aforementioned controversies and shed light on better pre-clinical planning.

The Finite element (FE) method has been widely used to evaluate stent behaviors [27–29] and predict vessel damage and adaptations [12–17]. Specifically, three-dimensional patient-specific artery models have been developed by coregistrating both optical coherence tomography (OCT) and computerized tomography (CT) images for performing structural and hemodynamic analyses [30]. It has been shown that the pre-dilatation and lesion compositions, such as lipid pool, fibrous cap, and calcification, affect the arterial responses [20, 21]. The abnormally high arterial stresses, induced by the stent implantation, is positively correlated with the risk of restenosis [18, 19]. Using patient-specific artery models, our group has systematically inspected the influence of the calcification attributes on the stenting outcomes, including stent underexpansion and malapposition [22, 23]. Our FE results have illustrated that a larger calcification angle constricted the stretchability of the lesion, and thus the stent expansion capacity. An in-vitro uniaxial tensile experiment also demonstrated that a larger calcification volume leads to a reduced stretchability of the lesion [25]. We conducted additional ex-vivo and in-silico experiments to further test this indicator in the post-dilation procedures [26]. These observations directed our attention to the stretchability of the lesion, i.e., the amount of fibrous plaque along the circumferential direction, which might be the the major determinant of stent expansion. With these accumulated studies on the correlation between lesion features and stenting outcomes, now it’s necessary to link the stenting outcomes in varied lesions with the required pressure for optimal stenting. Even a inflation pressure ranges from 10 to 20 atms has been adopted in the clinical practice, the amount of pressure load directly to the lesion is still a myth. Considering a pressure value of 6 atm has been widely adopted in the application of cutting beloon, the logic of the balloon design for different lesions has attracted more attentions. The quantifications of load transfer characteristics during stenting and post-dilation procedures are in immediate needs for enhanced understandings of the stent-artery interaction and designing better clinical strategies.

The objective of this work is to quantify the load sharing among the balloon, stent, artery and lesion components (i.e., fibrous plaque, and calcification, if any), during stenting and post-dilation in non-calcified and heavily calcified coronary arteries. The pressure-diameter relationship of the balloon, and the radial force transferred to the balloon, stent and lesion will be characterized. Further, the efficacy of high-pressure postdilation balloon will be inspected in terms of strain energy in each component and the maximum principal stresses in the artery. The fundamental understanding of load transfer and load sharing could better guide the optimal stenting in complex lesions.

## Materials and methods

### Model construction.

**Figure 1:**
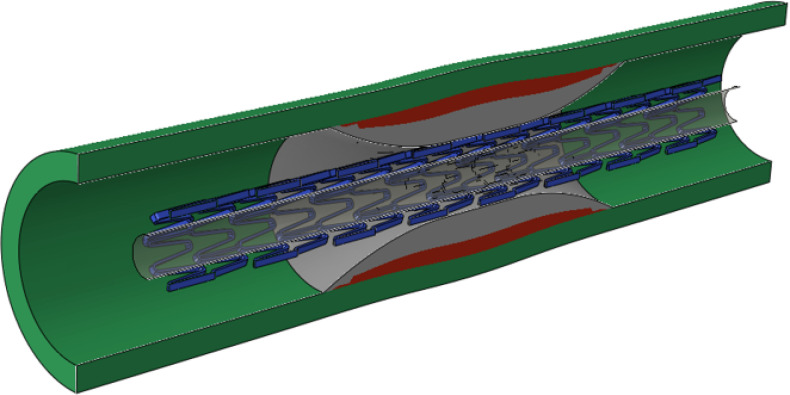
Geometric model of the heavily calcified coronary artery with a crimped Express stent. The fibrous plaque existed between calcification and artery.

An idealized coronary artery was modeled as a cylinder with a length of 20 mm, an inner diameter of 3 mm, and a thickness of 0.5 mm [31]. The center-located plaque had a parabolic shape with a length of 8.5 mm and a minimum diameter of 1.2 mm, mimicking a diameter stenosis of 60% (i.e., area stenosis of 84%). The fibrous plaque was adopted for the case of the non-calcified lesion. For the case of the heavily calcified lesion, a block calcification over a thin fibrous plaque was adopted, as shown in Fig. 1. The superficial concentric calcification had a maximum thickness of 0.64 mm, and the fibrous plaque had a maximum thickness of 0.26 mm. A commercial Express stent (Boston Scientific, Natick, MA, USA) was used. It has a length of 16 mm, a thickness of 0.13 mm and a nominal diameter of 3 mm at the nominal pressure of 12 atm. Non-compliant (NC) balloons with a fixed length of 17 mm, and diameters of 3 mm, 3.5 mm and 4mm, were simulated to study the impact of balloon oversizing. Additional NC balloons with a fixed nominal diameter of 3 mm, and lengths of 11 mm and 8.5 mm were also simulated to determine the efficacy of shortened balloons during post-dilation procedures. All NC balloons were simulated as cylinders with an initial diameter of 0.8 mm.

### Material properties.

The hyperelastic behaviors of the artery, fibrous plaque, and calcification were described by the reduced third-order polynomial strain energy density function U:

1 $$U=\sum_{i,j=1}^{3}\;C_{ij}{\left(I_{1}-3\right)}^{i}{\left(I_{2}-3\right)}^{j}$$

2 $$I_{1}=\lambda_{1}^{2}+\lambda_{2}^{2}+\lambda_{3}^{2}$$

3 $$I_{2}=\lambda_{1}^{-2}+\lambda_{2}^{-2}+\lambda_{3}^{-2}$$

**Figure 2:**
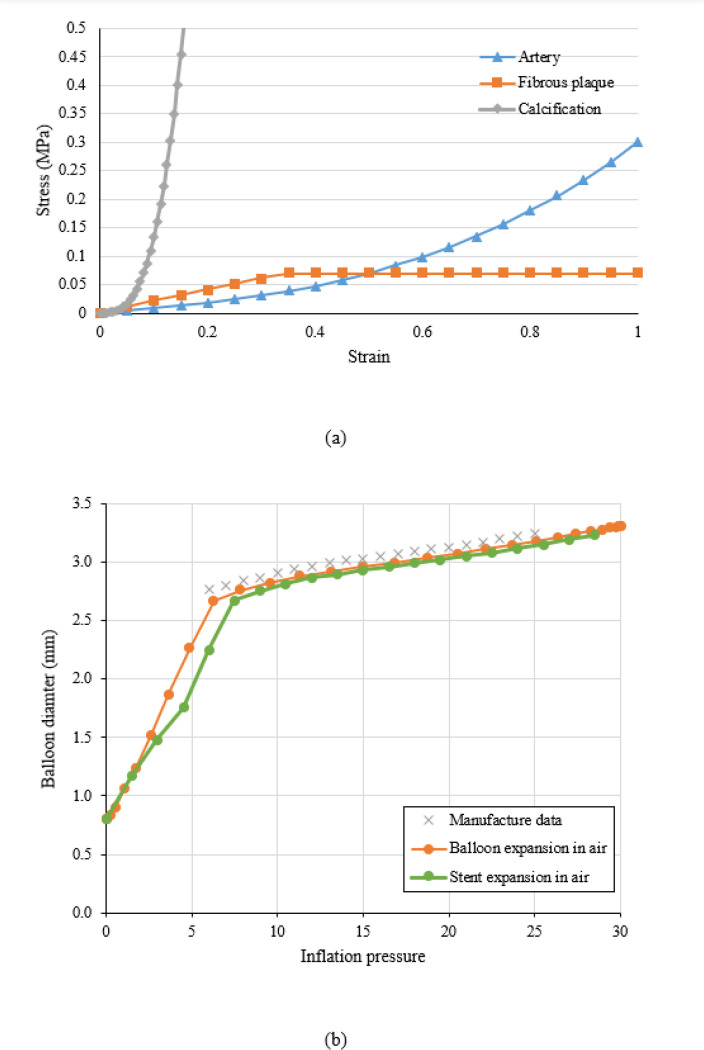
(a) Material properties of the artery, fibrous plaque, and calcification; (b) Pressure-diameter relationship between manufacturer data and simulation (balloon expansion, and stent expansion in the air).

The material coefficients $C_{ij}$ were adopted from our previous work [32], as shown in Table 1. A perfect plastic model was used to describe the tissue compaction of the fibrous plaque, which is necessary to capture a realistic stent expansion including stent recoil [33]. The plasticity of fibrous plaque was initiated at a strain of 34% when its stress reached its yield strength of 0.07MPa [34]. The stress-strain relationship for all these lesion components is shown in Fig. 2a.

The pressure-diameter data provided by the manufacturer was used to derive the material properties of the balloon (Fig. 2b). It is clear that the balloon exhibited a bilinear inflation behavior. The balloon diameter increased faster when the inflation pressure is below 6 atm, and much slower when the pressure exceeded 6 atm. To convert this bilinear inflation behavior of the balloon to a stress-strain relationship, the hoop strain was calculated as the relative change in diameter, and the hoop stress of a thin-walled cylinder was used to estimate the wall stress:

5 $$\epsilon =\frac{D-D_{0}}{D_{0}}$$

6 $$\sigma =\frac{PD}{2\delta }$$

where D is the diameter during expansion, $D_{0}$ is the initial diameter of 0.8 mm, P is the inflation pressure, and δ is the thickness of the balloon. The pressure-diameter curve obtained from simulation was compared with the manufacturing data with maximum difference less than 5% for pressure interval from 6 atm to 25 atm (Fig. 2b).

Finite element simulations of the calcified coronary artery in the context of idealized or patient-specific models have been well validated in our previous work by matching the stented lumen area with ex-vivo experiments, or matching the simulated stress level with the published data [22, 23, 26]. Mesh convergence studies were conducted in this work, and 123,100 hexahedron elements were adopted for the artery model. Symmetric boundary conditions (i.e., the displacement along the longitudinal direction is constrained, while along the transverse direction allowed) were applied to both ends of the artery and balloon such that the stenting procedure does not alter the lesion length far from the implantation site. For the stenting procedure, 10 atm was applied to the inner surface of the balloon. For the post-dilation, three pressures (i.e., 10 atm, 20 atm, and 30 atm) were sequentially applied to the inner surface of the balloon. General frictionless contacts were used for all interacting surfaces [35]. Energies were monitored during the stenting and post-dilation procedures to ensure the dynamic effect (i.e., inertial forces) was acceptable. The ratio of the kinetic energy to the internal energy of the whole model was kept below 5%. The models were solved using Abaqus/Explicit 2022 (Dassault Systemes Simulia Corp., Providence, RI, USA).

Following simulation, the load transfer and load sharing were quantified. The load transfer refers to the action-reaction force between balloon, stent, and lesions. The load sharing refers to the strain energy stored in each component of the lesion and the stent. The pressure load is calculated as the radial force applied to the inner surface of the balloon:

(7) $$F_{r}=P\pi DL$$

where P is the inflation pressure, D is the diameter of the balloon, and L is the length of the balloon. The radial forces applied on the inner surface of the stent and artery were obtained by adding all the radial component of the contact force at each node on their inner surface.

## Results

Stenting in both non-calcified and heavily calcified lesions was compared. The load transfer from inflation pressure to the balloon, stent, and artery were quantified. The stress analysis and load sharing among the balloon, stent, and artery were used to further quantify the efficacy of the high-pressure balloon in improving the stent expansion in heavily calcified lesion.

### Stent expansion and pressure-diameter curve.

**Figure 3:**
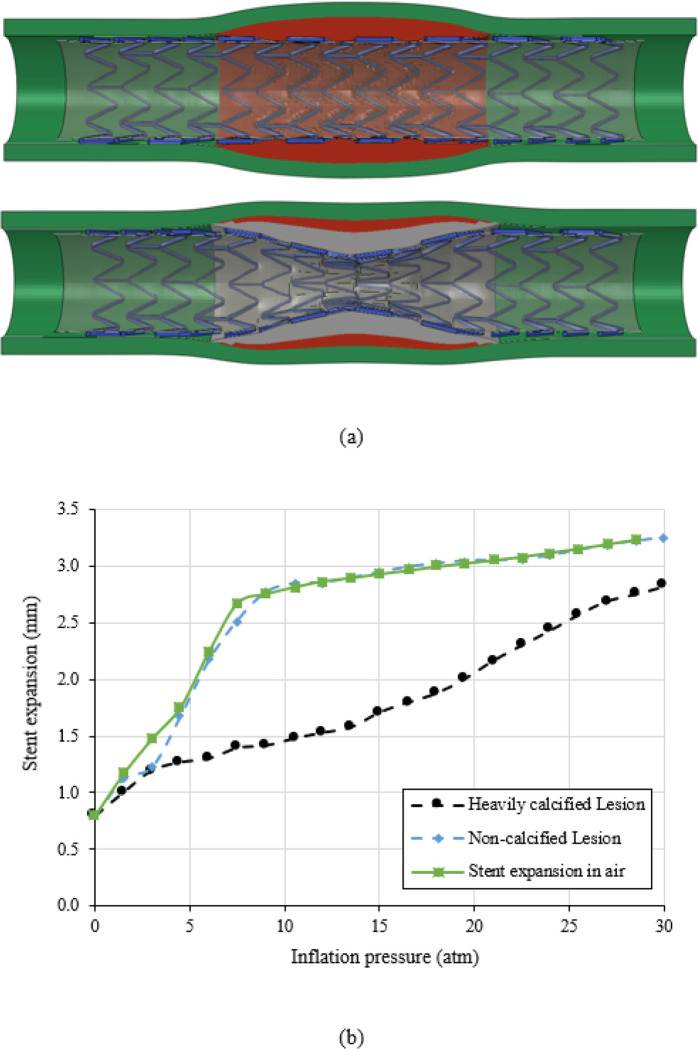
(a) Stent expansion profile in non-calcified (top) and heavily calcified (bottom) lesion; (b) Pressure-diameter relationship for stent expansion in the air, non-calcified lesion, and heavily calcified lesion.

The stent expansion in the non-calcified lesion and heavily calcified lesion are shown in Fig. 3. As inflation pressure increased to 10 atm, the stent expansion in the non-calcified lesion reached an inner diameter of 2.80 mm in the non-calcified artery, with a nearly cylindrical shape (Fig. 3a top). Stent underexpansion was observed in the heavily calcified artery, forming a dog bone shape with a minimal diameter of 1.48 mm (Fig. 3a bottom), lead to a residual stenosis of 49% in diameter and 74% in area. The associated pressure-diameter relationship is shown in Fig. 3b. The stent expansion in the non-calcified lesion was quite similar to the expansion in air, indicating the non-calcified lesion has minimal resistance and load sharing. On the contrary, the stent expansion in the heavily calcified lesion requires a much higher pressure beyond the normal pressure of 10 atm to obtain the predefined minimal diameter. As the inflation pressure increased from 10 atm to 30 atm, the minimal diameter of the non-calcified lesion increased from by only 19% (2.8 mm to 3.2 mm), while of the heavily calcified lesion it increased by 90% (from 1.48 mm to 2.82 mm).

### Radial force transferred from inflation pressure to balloon, stent, and lesion.

**Figure 4:**
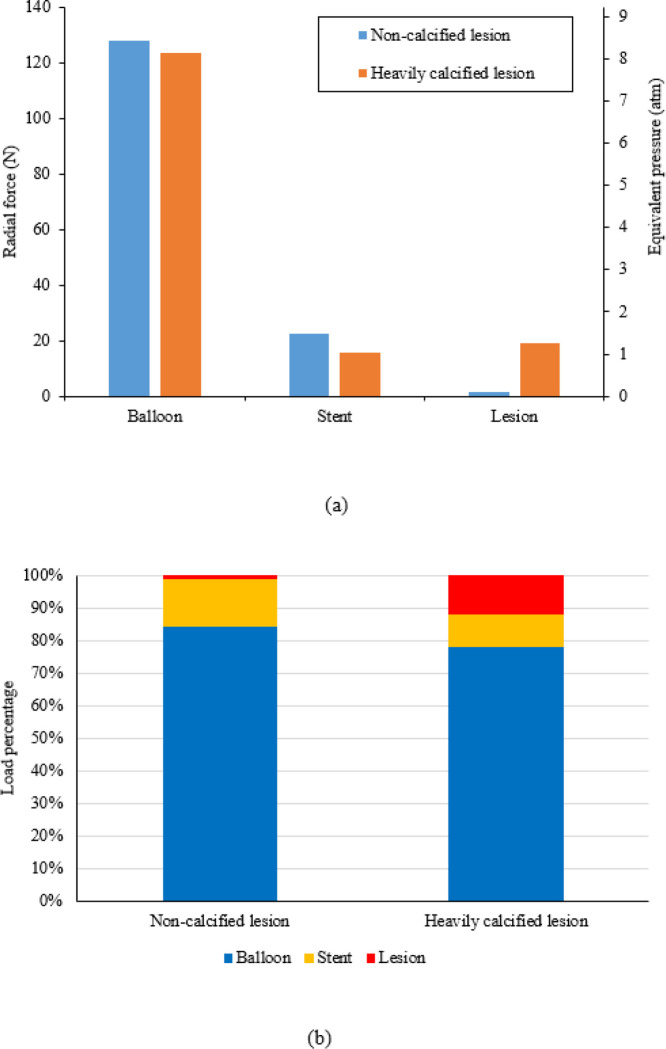
Radial force transferred from inflation pressure to the balloon, stent, and lesion in cases of stent expansion in non-calcified and heavily calcified lesions: (a) Clustered columns of radial force and equivalent pressure; (b) Stacked columns of pressure load percentage

The radial force transferred from the inflation pressure to the balloon, stent, and lesion are depicted in Fig. 4. The inflation pressure of 10 atm (i.e., 1.013 MPa) resulted in a cylindrical expansion with an inner diameter of 2.76 mm (Fig. 3), corresponding to a pressure load of 152 N exerted onto the balloon surface. For stent expansion in the non-calcified lesion, a radial force of 23.8 N (i.e., 15% of the pressure load) was transferred to the stent and lesion, of which 1.4 N (i.e., 0.8% of the pressure load) was transferred to the non-calcified lesion. For stent expansion in the heavily calcified lesion, a radial force of 34.5 N (i.e., 22% of the pressure load) was transferred to the stent and lesion, and 19 N (i.e., 12% of the pressure load) was transferred to the lesion. We could convert the radial force transfeerred to the lesion to an equivalent pressure using Eq. 7. Therefore, a pressure of 0.092 atm (i.e.,70 mmHg) was transferred to the non-calcified lesion, while a pressure of 1.25 atm (i.e., 950 mmHg) was transferred to the heavily calcified lesion. It should be noted that all the above load calculation is based on cylindrical shape assumption. Considering the actual dog-bone shape in the case of the heavily calcified lesion, the pressure load was less than 152 N, but the difference was less than 10%.

### Load sharing in terms of strain energy during stenting and post-dilations.

**Figure 5:**
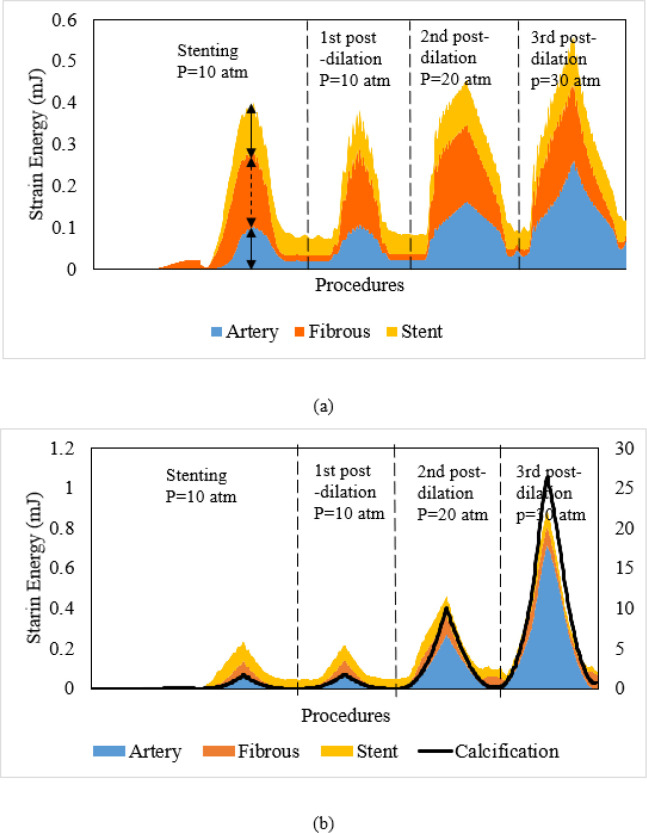
Stacked area graph of strain energy following stenting and post-dilations in (a) non-calcified lesion and (b) heavely calcified lesion. Right axis is for calcification curve only.

Strain energy stored in the artery, fibrous plaque, calcification (if any), and stent during the stenting and post-dilation procedures are shown in Fig. 5. The stacked areas are used to show how each component contributes to the total strain energy of the stented lesion. It is clear that, regardless of the lesion type, the absorbed strain energy in all components reached its peak at each target inflation pressure, and then recoiled back following the balloon defaltion. For the non-calcified lesion, the peak strain energy of fibrous plaque was 0.184 mJ at the full expansion of the stent (arrow in Fig. 5a), and was 0.182 mJ, 0.184mJ, 0.189 mJ, respectively, during three sequential postdilations with increased inflation pressures. The peak strain energy stored in both stent and lesion was 0.35mJ during stenting and increased to 0.55 mJ during the 3rd post-dilation at the inflation pressure of 30 atm. In the heavily calcified lesion, the corresponding total strain energy stored in the stent, fibrous plaque and artery was 0.21 mJ during stenting and sharply increased to 0.90 mJ during the 3rd post-dilation at inflation pressure of 30 atm. The peak strain energy in the calcification alone (labeled in the right y-axis of Fig. 5b) was 1.77 mJ, and increased to 26.5 mJ (15 times) during the 3rd post-dilation at the inflation pressure of 30 atm, making it substancially higher than the other components. The huge increase of the strain energy in calcification indicate a potential of fracture.

### Maximum principal stresses induced in lesions after stenting and post-dilations.

**Figure 6:**
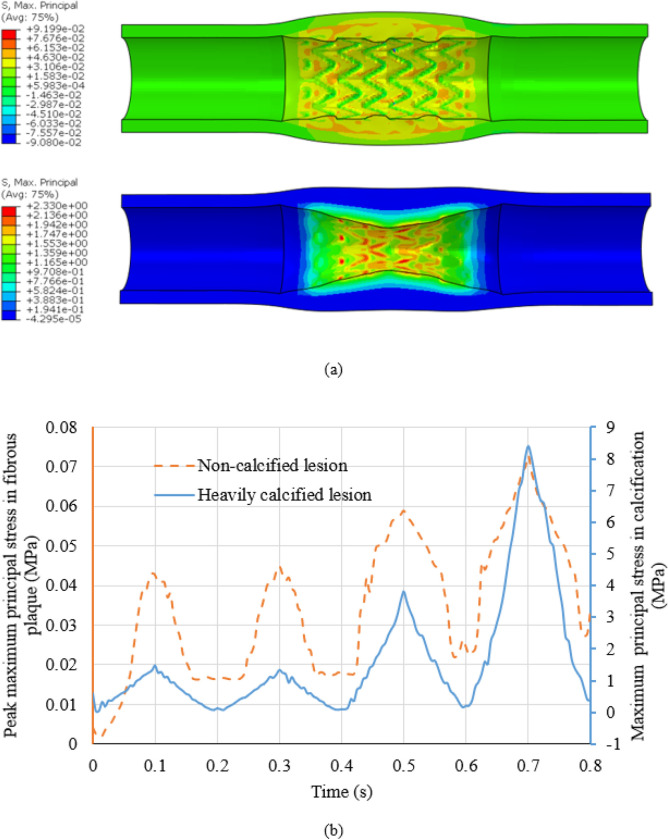
(a) Direct stenting induced maximum principal stress (unit: MPa) maps in non-calcified (top) and heavily calcified lesions (bottom) at the balloon pressure of 10 atm; (b) The peak maximum principal stress of plaque for stent expansion in non-calcified lesion case (orange) and heavily calcified lesion case (blue) during stenting and three sequential post-dilations at pressure of 10, 20 and 30 atm.

The distribution of maximum principal stress (MPS) on the lesions at 10 atm was depicted in Fig. 6a. The time history of the peak value of MPS in the non-calcified and heavily calcified lesions during stenting, and the three sequential post-dilations at pressures of 10, 20 and 30 atm, were plotted in Fig. 6b. The peak value of MPS in the non-calcified lesion, occurred in the artery, was 40 kPa during stenting at a inflation pressure of 10 atm, and increased to 70 kPa during the 3rd post-dilation at a inflation pressure of 30 atm. For the heavily calcified lesion, the peak value of MPS, occurred in the calcification, was 1.5 MPa during stenting at a inflation pressure of 10 atm, and increased to 8.4 MPa during the 3rd post-dilation at a inflation pressure of 30 atm. It is worth noting that the MPS time history is consistent with the load sharing analysis (Fig. 5). For the non-calcified lesion, the arterial tissue only stores a small portion of energy and doesn’t increase proportionally along with the increase of the inflation pressure. This indicates a lower risk of vessel rupture. On the contrary, strain energy stored in the calcification increase 14 times as the inflation pressure increase from 10 atm to 30 atm, indicateing a higher probability of calcification fracture.

## Discussion

Suboptimal stenting (stent underexpansion, malapposition etc.) in complex lesions, especially in heavily calcified lesions, has gained increasing attentions. Plaque modification and/or post-dilation with a high-pressure balloon has been developped to improve the stent expansion and apposition. The cardiologists are under a pressure to choose efficient procedures for a target stent expansion with less risk of vessel rupture. In this work, the link between the inflation pressure to the exact force exerted on the inner surface of various lesions was inspected with computer simulations. To our best knowledge, this is the first study to quantify the load transfer between stent-artery interaction and load-sharing capacities of different components of the during stenting and post-dilation procedures. Results have emphasized the monitoring the stent expansion during stenting and supported the design of the high pressure balloon. The stent expansion showed a nearly cylindrical shape in the non-calcified lesion and the balloon undertook most of the increasing inflation pressure load. On the contrast, stent expansion showed a dog boen shape in the heavily calcified lesion, and the increasing inflation pressure can effectively increase the minimal lumen area, stress in the calcification, and potential of calcification fracture. The efficacy of the increasing inflation pressure in the heavily calcifcaiton lesion is due to the dog bone shape of the stent expansion.

Most previous computational studies of stent-artery interactions focused on the mechanical environment change (i.e., stress and strain) in the arterial tissues [20, 35] or stent fracture when exposed to cardiac wall movement following stenting [36]. Our previous works have investigated the influence of calcification attributes on stenting expansion with stylized and patient specific artery models [22, 37], and results have shown that the calcification will reduce the stretch capability of the lesion, further leading to stent underexpansion. In this work, load transfer analyses were conducted for stenting in non-calcified and heavily calcified lesions to quantify the effective load for stent expansion. Only a small portion of the pressure load (1.4 N, 0.8% of the total pressure load) was transferred to the non-calcified lesion. This small portion of the pressure load only induced an effective pressure of 75 mmHg, which is slightly less than the tested blood pressure in a human body, which is around 100 mmHg [38]. Additionally, the balloon pressure for stenting, which is usually around 12 atm (or 9120 mmHg), is much higher than the blood pressure. This drastic difference between the dilation pressure and the exact pressure required for expanding the lesion has previously been ignored in studies on stent-artery interactions. The load transfer and load sharing analysis will build a direct link between the increasing pressure and the effective force to the lesion, which will provide a rational index for optimal stenting. In addition, a radial force of 22.4 N was required to expand the stent, which is similar to the reported studies [39, 40]. These agreements validate the feasibility of our model and methods. For the heavily calcified lesion, the concentrated, thick calcification acts as a stiff ring and causes the observed dog bone shape of the stent expansion. The balloon which expands in a dog bone shape was not able to undertake as high of a pressure load as the cylindrical shaped balloon. Because of this, a higher-pressure load of 19 N (1027 mmHg) was transferred to the lesion, which is more than 12 times that which is shown in the non-calcified lesion. We can image that as the lesion changes from non-calcified to a mild, medium, and then heavily calcified lesion, the balloon stent expansion will change from a nearly cylindrical shape to a more irregular and/or dog bone shape, during which the pressure load transferred to the lesion will increase from a lower to a higher value. Our previous virtual bench tests also have shown that the resultant contact along the normal of one cut plane increased from 1.5 N to 2.5 N, to 3.5 N as the calcification angle increased from 60°, to 180°, to 270° [41].

Post-dilation has long been adopted to improve stent expansion by increasing inflation pressure or using a larger balloon. One study has even shown that high pressures up to 40 atm can lead to an improved stent deployment in heavily calcified lesions [3]. Some other retrospective studies, however, have showed that a pressure of 16 atm and a balloon diameter which is 0.5 mm larger than the nominal diameter of stent are two critical cap values, and there will be more complications if the inflation pressure exceeds these [4]. In this work, the load sharing analysis for post-dilation with increasing pressure was done to evaluate the efficacy of the procedure. In the non-calcified lesion, the stent expansion has a nearly cylindrical profile (diameter close to manufacturing data) and the balloon undertook most of the pressure load. Therefore, the increasing pressure didn’t significantly increase the minimal lumen diameter, stress or strain energy in the lesion. For the heavily calcified lesion, the stent expansion has a dog bone shape and the increasing pressure load can effectively increase the minimal lumen diameter, stress and strain anergy in the calcification, and the potential of calcification fracture. Based on this observation, increasing pressure can be an effective procedure to improve the stent expansion if a dog boen shape was observed. In addition, a larger balloon may cause the lesion to rupture if the calcification suddenly fractures at a high pressure, which may lead to acute myocardial infarction as reported in clinics [2]. These findings indicate that the monitoring of the stenting process, especially the stenting profile and diameter at the target inflation pressure, may help in making optimal stenting decisions for patients. Our results also support the design and application of the high pressure ballon. Currently, the burst pressure of most post-dilaiton balloon is around 20 atm, while it can not induce a calcification fracture, and the cardiologists shift to alergar balloon, with the risk of vessel rupture. As we noticed that the increasing pressure can effective exert more force to the lesion, the high pressure balloon can be a safe way to induce the calcification fracture by increasing the inflation pressure to a higher pressure limit of the balloon.

There are some limitations in this work, such as the selection of material model and simulation techniques, as well as simplifications to the overall testing systems and assumptions. The isotropic hyperelastic models were adopted to describe the mechanical behavior of the arterial tissue without considering the anisotropic or viscoelastic behaviors. The plaque was simplified into two materials: fibrosis and calcification. The complexity and eccentricity of the lesion were also simplified as the symmetric model for comparison with the analytical results. The balloon characteristics were also simplified to help better understanding of the load transfer. These simplifications may cause a deviation in the stress analyses, but won’t affect the global values such as the resulted force or strain energy, so the conclusions in this work can still be applied for a model with further specificity. The fracture behavior of the calcification was not considered in this work since our focus is the load sharing analysis to provide the fundamental understanding, the fracture behavior will affect the evolution of the restored strain energy. We simulated the balloon with a cylindrical surface, rather than the three-folded balloon based on our benchtop test in which the balloon exhibited a cylindrical shape even at very low pressure (1 atm). Once the inflation pressure exceeded the nominal pressure, the balloon showed a larger resistance. We captured the bilinear behavior of the balloon and verified it with the manufacturer data. The findings from this work will be further validated with clinical observations.

## Conclusion

In this work, the diameter-pressure curve and load transfer analysis for stenting in non-calcified and heavily calcified coronary artery were performed. Further load sharing analysis and stress analysis were conducted to investigate the efficacy of increasing the pressure over improving the stenting expansion in heavily calcified coronary artery. The stent expansion showed a nearly cylindrical shape in the non-calcified lesion and the balloon undertook most of the increasing inflation pressure load. On the contrast, stent expansion showed a dog boen shape in the heavily calcified lesion, and the increasing inflation pressure can effectively increase the minimal lumen area, stress in the calcification, and potential of calcification fracture. This study suggested that monitoring the stenting process with angiography imaging, especially a precise stent expansion profile at the target inflation pressure will help optimize the stenting procedure in complex lesions. Our results also support the design and application of the high pressure balloon for post dilation.

## Figures and Tables

**Table 1:** Material coefficients

	C_10_(MPa)	C_01_(MPa)	C_11_(MPa)	C_20_(MPa)	C_02_(MPa)	C_30_(MPa)	C_03_(MPa)
Artery	0.10881	−0.101	−0.1790674	0.0885618	0.062686		
Fibrous plaque	0.04				0.003		0.02976
Calcification	−0.49596	0.50661	1.19353	3.6378		4.73725	

## Data Availability

The datasets used and/or analysed during the current study available from the corresponding author on reasonable request.

## References

[1] Seth A., Gupta S., Pratap Singh V., and Kumar V., 2017, “Expert Opinion: Optimising Stent Deployment in Contemporary Practice: The Role of Intracoronary Imaging and Non-Compliant Balloons,” Interv Cardiol, 12(2), pp. 81–84. 29588734 10.15420/icr.2017:12:1 PMC5808472

[2] Zhang Z.-J., Marroquin O. C., Stone R. A., Weissfeld J. L., Mulukutla S. R., Selzer F., and Kip K. E., 2010, “Differential Effects of Post-Dilation after Stent Deployment in Patients Presenting with and without Acute Myocardial Infarction,” Am Heart J, 160(5), pp. 979–986.e1. 21095289 10.1016/j.ahj.2010.07.007 PMC3003443

[3] Díaz J. F., Gómez-Menchero A., Cardenal R., Sánchez-González C., and Sanghvi A., 2012, “Extremely High-Pressure Dilation with a New Noncompliant Balloon,” Tex Heart Inst J, 39(5), pp. 635–638. 23109756 PMC3461667

[4] Ortega-Paz L., Capodanno D., Gori T., Nef H., Latib A., Caramanno G., Di Mario C., Naber C., Lesiak M., Capranzano P., Wiebe J., Mehilli J., Araszkiewicz A., Pyxaras S., Mattesini A., Geraci S., Naganuma T., Colombo A., Münzel T., Sabaté M., Tamburino C., and Brugaletta S., 2017, “Predilation, Sizing and PostDilation Scoring in Patients Undergoing Everolimus-Eluting Bioresorbable Scaffold Implantation for Prediction of Cardiac Adverse Events: Development and Internal Validation of the PSP Score,” Eurolntervention, 12(17), pp. 2110–2117. 10.4244/EIJ-D-16-00974 28246060

[5] Brueck M., Scheinert D., Wortmann A., Bremer J., Korn H. von, Klinghammer L., Kramer W., Flachskampf F. A., Daniel W. G., and Ludwig J., 2002, “Direct Coronary Stenting versus Predilatation Followed by Stent Placement,” American Journal of Cardiology, 90(11), pp. 1187–1192. 12450596 10.1016/s0002-9149(02)02832-1

[6] Kocum T., Yurtdas M., Ozcan T., Akcay B., Erol T., Camsari A., and Doven O., 2008, “Direct Stenting versus Predilatation and Stenting Technique When Using Paclitaxel-Eluting Stents,” Int Heart J, 49(5), pp. 545–552. 18971566 10.1536/ihj.49.545

[7] Kovar L. I., Monrad E. S., Sherman W., Kunchithapatham S., Ravi K. L., Gotsis W., Silverman G., and Brown D. L., 2001, “A Randomized Trial of Stenting with or without Balloon Predilation for the Treatment of Coronary Artery Disease,” Am Heart J, 142(5), p. E9. 11685184 10.1067/mhj.2001.119124

[8] Yamamoto Y., Kawarada O., Ando H., Anzai H., Zen K., Tamura K., Tsukahara K., Tsubakimoto Y., Toma M., Nakamura S., Nakamura H., Hozawa K., Yokoi Y., and Yasuda S., 2020, “Effects of High-Speed Rotational Atherectomy in Peripheral Artery Disease Patients with Calcified Lesions: A Retrospective Multicenter Registry,” Cardiovasc Interv and Ther, 35(4), pp. 393–397. 32112238 10.1007/s12928-020-00643-9

[9] 2015, “Long-Term Safety and Performance of the Orbital Atherectomy System for Treating Calcified Coronary Artery Lesions: 5-Year Follow-up in the ORBIT I Trial,” Cardiovascular Revascularization Medicine, 16(4), pp. 213–216. 25866032 10.1016/j.carrev.2015.03.007

[10] Sotomi Y., Shlofmitz R. A., Colombo A., Serruys P. W., and Onuma Y., 2016, “Patient Selection and Procedural Considerations for Coronary Orbital Atherectomy System,” Interv Cardiol, 11(1), pp. 33–38. 29588702 10.15420/icr.2015:19:2 PMC5808671

[11] Chambers Jeffrey W., Feldman Robert L., Himmelstein Stevan I., Rohit Bhatheja, Villa Augusto E., Strickman Neil E., Shlofmitz Richard A., Dulas Daniel D., Dinesh Arab, Khanna Puneet K., Lee Arthur C., Ghali Magdi G.H., Shah Rakesh R., Davis Thomas P., Kim Christopher Y., Zaheed Tai, Patel Kirit C., Puma Joseph A., Prakash Makam, Bertolet Barry D., and Nseir Georges Y., 2014, “Pivotal Trial to Evaluate the Safety and Efficacy of the Orbital Atherectomy System in Treating De Novo, Severely Calcified Coronary Lesions (ORBIT II),” JACC: Cardiovascular Interventions, 7(5), pp. 510–518. 24852804 10.1016/j.jcin.2014.01.158

[12] Laubrie J. D., Mousavi J. S., and Avril S., 2020, “A New Finite-Element Shell Model for Arterial Growth and Remodeling after Stent Implantation,” International Journal for Numerical Methods in Biomedical Engineering, 36(1), p. e3282. 31773919 10.1002/cnm.3282

[13] Zheng Q., Dong P., Li Z., Lv Y., An M., and Gu L., 2020, “Braided Composite Stent for Peripheral Vascular Applications,” Nanotechnology Reviews, 9(1), pp. 1137–1146. 35936942 10.1515/ntrev-2020-0056 PMC9354498

[14] Lin S., Dong P., Zhou C., Dallan L. A. P., Zimin V. N., Pereira G. T. R., Lee J., Gharaibeh Y., Wilson D. L., Bezerra H. G., and Gu L., 2020, “Degradation Modeling of Poly-I-Lactide Acid (PLLA) Bioresorbable Vascular Scaffold within a Coronary Artery,” Nanotechnology Reviews, 9(1), pp. 1217–1226. 34012762 10.1515/ntrev-2020-0093 PMC8130847

[15] Wang R., Zuo H., Yang Y.-M., Yang B., and Li Q., 2017, “Finite Element Simulation and Optimization of Radial Resistive Force for Shape Memory Alloy Vertebral Body Stent,” Journal of Intelligent Material Systems and Structures, 28(15), pp. 2140–2150.

[16] Zheng Q., Dong P., Li Z., Han X., Zhou C., An M., and Gu L., 2019, “Mechanical Characterizations of Braided Composite Stents Made of Helical Polyethylene Terephthalate Strips and NiTi Wires,” Nanotechnology Reviews, 8(1), pp. 168–174. 35966892 10.1515/ntrev-2019-0016 PMC9368628

[17] 2018, “Migration Resistance of Esophageal Stents: The Role of Stent Design,” Computers in Biology and Medicine, 100, pp. 43–49. 29975854 10.1016/j.compbiomed.2018.06.031

[18] Gu L., Zhao S., Muttyam A. K., and Hammel J. M., 2010, “The Relation Between the Arterial Stress and Restenosis Rate After Coronary Stenting,” Journal of Medical Devices, 4(031005).

[19] 2005, “Cardiovascular Stent Design and Vessel Stresses: A Finite Element Analysis,” Journal of Biomechanics, 38(8), pp. 1574–1581. 15958213 10.1016/j.jbiomech.2004.07.022

[20] Wei L., Chen Q., and Li Z., 2019, “Influences of Plaque Eccentricity and Composition on the Stent–Plaque-Artery Interaction during Stent Implantation,” Biomech Model Mechanobiol, 18(1), pp. 45–56. 30097815 10.1007/s10237-018-1066-z

[21] Conway C., McGarry J. P., Edelman E. R., and McHugh P. E., 2017, “Numerical Simulation of Stent Angioplasty with Predilation: An Investigation into Lesion Constitutive Representation and Calcification Influence,” Ann Biomed Eng, 45(9), pp. 2244–2252. 28488215 10.1007/s10439-017-1851-3 PMC5963267

[22] Dong P., Bezerra H. G., Wilson D. L., and Gu L., 2018, “Impact of Calcium Quantifications on Stent Expansions,” Journal of Biomechanical Engineering, 141(021010). 10.1115/1.4042013 PMC6298533 30453326

[23] Dong P., Mozafari H., Prabhu D., Bezerra H. G., Wilson D. L., and Gu L., 2020, “Optical Coherence Tomography-Based Modeling of Stent Deployment in Heavily Calcified Coronary Lesion,” Journal of Biomechanical Engineering, 142(051012). 10.1115/1.4045285 PMC7104774 31654052

[24] Dong P., Ye G., Kaya M., and Gu L., 2020, “Simulation-Driven Machine Learning for Predicting Stent Expansion in Calcified Coronary Artery,” Applied Sciences, 10(17), p. 5820. 35903558 10.3390/app10175820 PMC9328568

[25] Barrett H. E., Cunnane E. M., Hidayat H., O Brien J. M., Kavanagh E. G., and Walsh M. T., 2017, “Calcification Volume Reduces Stretch Capability and Predisposes Plaque to Rupture in an in Vitro Model of Carotid Artery Stenting,” Eur J Vasc Endovasc Surg, 54(4), pp. 431–438. 28838637 10.1016/j.ejvs.2017.07.022

[26] Dong P., Mozafari H., Lee J., Gharaibeh Y., Zimin V. N., Dallan L. A. P., Bezerra H. G., Wilson D. L., and Gu L., 2021, “Mechanical Performances of Balloon Post-Dilation for Improving Stent Expansion in Calcified Coronary Artery: Computational and Experimental Investigations,” Journal of the Mechanical Behavior of Biomedical Materials, 121, p. 104609. 34082181 10.1016/j.jmbbm.2021.104609

[27] Schiavone A., and Zhao L. G., 2015, “A Study of Balloon Type, System Constraint and Artery Constitutive Model Used in Finite Element Simulation of Stent Deployment,” Mechanics of Advanced Materials and Modern Processes, 1(1), p. 1.

[28] Gervaso F., Capelli C., Petrini L., Lattanzio S., Di Virgilio L., and Migliavacca F., 2008, “On the Effects of Different Strategies in Modelling Balloon-Expandable Stenting by Means of Finite Element Method,” J Biomech, 41(6), pp. 1206–1212. 18374340 10.1016/j.jbiomech.2008.01.027

[29] De Beule M., Mortier P., Carlier S. G., Verhegghe B., Van Impe R., and Verdonck P., 2008, “Realistic Finite Element-Based Stent Design: The Impact of Balloon Folding,” J Biomech, 41(2), pp. 383–389. 17920068 10.1016/j.jbiomech.2007.08.014

[30] 2016, “Computational Replication of the Patient-Specific Stenting Procedure for Coronary Artery Bifurcations: From OCT and CT Imaging to Structural and Hemodynamics Analyses,” Journal of Biomechanics, 49(11), pp. 2102–2111. 26655589 10.1016/j.jbiomech.2015.11.024

[31] Martin D., and Boyle F., 2013, “Finite Element Analysis of Balloon-Expandable Coronary Stent Deployment: Influence of Angioplasty Balloon Configuration: FINITE ELEMENT ANALYSIS OF CORONARY STENT DEPLOYMENT,” Int. J. Numer. Meth. Biomed. Engng., 29(11), pp. 1161–1175. 10.1002/cnm.2557 23696255

[32] Zhao S., Gu L., and Froemming S. R., 2012, “Finite Element Analysis of the Implantation of a SelfExpanding Stent: Impact of Lesion Calcification,” Journal of Medical Devices, 6(021001).

[33] Liang D. K., Yang D. Z., Qi M., and Wang W. Q., 2005, “Finite Element Analysis of the Implantation of a Balloon-Expandable Stent in a Stenosed Artery,” International Journal of Cardiology, 104(3), pp. 314–318. 16186062 10.1016/j.ijcard.2004.12.033

[34] Gastaldi D., Morlacchi S., Nichetti R., Capelli C., Dubini G., Petrini L., and Migliavacca F., 2010, “Modelling of the Provisional Side-Branch Stenting Approach for the Treatment of Atherosclerotic Coronary Bifurcations: Effects of Stent Positioning,” Biomech Model Mechanobiol, 9(5), pp. 551–561. 20155479 10.1007/s10237-010-0196-8

[35] Pericevic I., Lally C., Toner D., and Kelly D. J., 2009, “The Influence of Plaque Composition on Underlying Arterial Wall Stress during Stent Expansion: The Case for Lesion-Specific Stents,” Medical Engineering & Physics, 31(4), pp. 428–433. 19129001 10.1016/j.medengphy.2008.11.005

[36] Morlacchi S., Pennati G., Petrini L., Dubini G., and Migliavacca F., 2014, “Influence of Plaque Calcifications on Coronary Stent Fracture: A Numerical Fatigue Life Analysis Including Cardiac Wall Movement,” Journal of Biomechanics, 47(4), pp. 899–907. 24468208 10.1016/j.jbiomech.2014.01.007

[37] Dong P., Mozafari H., Prabhu D., Bezerra H. G., Wilson D. L., and Gu L., 2020, “Optical Coherence Tomography-Based Modeling of Stent Deployment in Heavily Calcified Coronary Lesion,” Journal of biomechanical engineering, 142(5). 10.1115/1.4045285 PMC7104774 31654052

[38] “Understanding Blood Pressure Readings,” www.heart.org [Online]. Available: https://www.heart.org/en/health-topics/high-blood-pressure/understanding-blood-pressure-readings. [Accessed: 24-Apr-2022].

[39] Gökgöl C., Diehm N., Nezami F. R., and Büchler P., 2015, “Nitinol Stent Oversizing in Patients Undergoing Popliteal Artery Revascularization: A Finite Element Study,” Ann Biomed Eng, 43(12), pp. 2868–2880. 26101031 10.1007/s10439-015-1358-8

[40] Kumar A., and Bhatnagar N., 2021, “Finite Element Simulation and Testing of Cobalt-Chromium Stent: A Parametric Study on Radial Strength, Recoil, Foreshortening, and Dogboning,” Computer Methods in Biomechanics and Biomedical Engineering, 24(3), pp. 245–259. 33021106 10.1080/10255842.2020.1822823

[41] Dong P., Lin S., Wilson D. L., Bezerra H. G., and Gu L., 2017, “Target Lesion Calcium Arc Influence the Performance of Stenting,” American Society of Mechanical Engineers Digital Collection. 10.1115/DMD2017-3455 PMC5906068 29683139

